# Genetic trends for yield and key agronomic traits in pre-commercial and commercial maize varieties between 2008 and 2020 in Uganda

**DOI:** 10.3389/fpls.2023.1020667

**Published:** 2023-03-10

**Authors:** Godfrey Asea, Daniel Bomet Kwemoi, Clay Sneller, Charles L. Kasozi, Biswanath Das, Lennin Musundire, Dan Makumbi, Yoseph Beyene, Boddupalli M. Prasanna

**Affiliations:** ^1^ National Crops Resources Research Institute, National Agricultural Research Organization, Kampala, Uganda; ^2^ Department of Horticulture and Crop Science, The Ohio State University, Wooster, OH, United States; ^3^ Global Maize Program, International Maize and Wheat Improvement Center (CIMMYT), Nairobi, Kenya

**Keywords:** maize breeding, genetic gains, maize yields and agronomic traits, regression analysis, productivity gains

## Abstract

Estimating genetic gains is vital to optimize breeding programs for increased efficiency. Genetic gains should translate into productivity gains if returns to investments in breeding and impact are to be realized. The objective of this study was to estimate genetic gain for grain yield and key agronomic traits in pre-commercial and commercial maize varieties from public and private breeding programs tested in (i) national performance trials (NPT), (ii) era trial and, (iii) compare the trends with the national average. The study used (i) historical NPT data on 419 improved maize varieties evaluated in 23 trials at 6-8 locations each between 2008 and 2020, and (ii) data from an era trial of 54 maize hybrids released between 1999 and 2020. The NPT data was first analyzed using a mixed model and resulting estimate for each entry was regressed onto its first year of testing. Analysis was done over all entries, only entries from National Agricultural Research Organization (NARO), International Maize and Wheat Improvement Center (CIMMYT), or private seed companies. Estimated genetic gain was 2.25% or 81 kg ha^-1^ year^-1^ from the NPT analysis. A comparison of genetic trends by source indicated that CIMMYT entries had a gain of 1.98% year^-1^ or 106 kg ha^-1^ year^-1^. In contrast, NARO and private sector maize entries recorded genetic gains of 1.30% year^-1^ (59 kg ha^-1^ year^-1^) and 1.71% year^-1^ (79 kg ha^-1^ year^-1^), respectively. Varieties from NARO and private sector showed comparable mean yields of 4.56 t ha^-1^ and 4.62 t ha^-1^, respectively, while hybrids from CIMMYT had a mean of 5.37 t ha^-1^. Era analysis indicated significant genetic gain of 1.69% year^-1^ or 55 kg ha^-1^ year^-1^, while a significant national productivity gain of 1.48% year^-1^ (37 kg ha^-1^ year^-1^) was obtained. The study, thus, demonstrated the importance of public-private partnerships in development and delivery of new genetics to farmers in Uganda.

## 1 Introduction

Maize (*Zea mays* L.) is an important staple food crop in sub-Sahara Africa (SSA), providing food security and income to more than 208 million households. It occupies the largest land area of all staples, with more than 35 million hectares (M ha) harvested annually (FAOSTAT, 2018). In Uganda, maize is an important staple crop serving the dual roles of food and feed. It is also a key crop that plays a crucial role in the national economy in providing employment along its value chain, household income, and revenue from significant exports to the East African region. It is estimated that approximately 3.6 million households (mainly smallholders) grow maize on more than 1.9 M ha (about 20% of the total crop area) (Uganda Bureau of Statistics (UBOS), 2022). Over the past 15 years, maize production and productivity have almost doubled, with gains arising mainly from increased adoption of improved varieties, expansion in the area, emerging commercial farmers, increased access to improved seed, and favorable regional grain trade policies. Current maize yield productivity in Uganda is estimated to be 2.5 t ha^-1^ with a total production of 4.6 million tonnes (Uganda Bureau of Statistics (UBOS), 2022). This increase in maize production and productivity has been realized despite the emergence of threats such as fall armyworm since 2016 (Otim et al., 2018) and maize lethal necrosis since 2012 (Mahuku et al., 2015; Prasanna et al., 2022a), limited fertilizer use, poorly structured grain markets, volatility in grain prices, limited farmer access to extension services, and quality inputs (Kilimo Trust, 2018). Breeding efforts to address the major biotic and abiotic stresses are ongoing in collaborative efforts between national agricultural research and extension systems (NARES), Consultative Group on International Agricultural Research (CGIAR) centers, and the private sector.

The fundamental change in maize breeding in sub-Saharan Africa occurred in the early 2000s with a particular focus on delivering stress-tolerant maize varieties to mitigate the impacts of climate change, especially frequent droughts and complex diseases (Pratt et al., 2004; Bäzinger et al., 2006; Kyetere et al., 2019; Prasanna et al., 2021). The orientation of breeding towards climate adaptation and resilience in the last decade has contributed to the successful development and release of multiple-stress tolerant maize varieties, with significant yield advantages over the market-dominant but obsolete varieties (Setimela et al., 2017; Simtowe et al., 2019; Prasanna et al., 2020). The breeding programs have been supported by increased investment in phenotyping capacity and extensive germplasm testing networks which have resulted in genetic gains in the region (Cairns et al., 2013; Masuka et al., 2017a; Masuka et al., 2017b; Cairns and Prasanna, 2018; Prasanna et al., 2021). The combined efforts of national, public and private sector maize breeding in Uganda have resulted in the release of 84 improved maize varieties between 2007 and 2020. The distribution of these variety releases by source is 41% from the private sector, 39% by CGIAR and 20% from the National Agricultural Research Organization (NARO) maize breeding programs. It is worth noting that CGIAR centers submit hybrids into NPTs through both the private sector and national programs. Promising hybrids nominated from breeding pipelines intended for commercial release are by law submitted to pre-commercial testing in National Performance Trials (NPTs) according to the established guidelines, ensuring DUS (Distinctness, Uniformity, Stability) and VCU (Value for Cultivation and Use). In Uganda, the National Seed Certification Services delegated the mandate of conducting NPTs to NARO, following established standards and protocols.

The seed industry is an important vehicle for delivering improved genetics and, plays an important role in the growth and evolution of the maize sector. The maize seed sector in Uganda is characterized by the co-existence of both the formal and informal systems, with the formal sector contributing less than 50% of the national seed requirements (Mabaya et al., 2021). The seed sector is instrumental in making the seed of the new and improved varieties available to the farmers, and has thus enabled increased yield and productivity. Despite increased maize production and productivity in Uganda, no studies have been conducted so far to quantify the genetic gains made over the years. Estimating genetic gains provides an opportunity to monitor the progress being made by the breeding programs in delivering better genetics to the farmers. In addition, genetic gain estimates help to monitor breeding efficiency and identify areas of improvement and investment for accelerated genetic gains. Era or legacy studies, whereby varieties released in different years are evaluated in the same trials, provide the most unbiased estimates of genetic gain because they avoid differences in agronomic management or climate variability, which can potentially confound the genetic trend (Rutkoski, 2019a; Rutkoski, 2019b). The breeder’s equation is often used to predict what gains may be achieved using estimates of parameters. However, it is important to estimate realized genetic gains from actual field trials, retrospectively (Prasanna et al., 2022). Therefore, the objective of this study was to assess the realized and historical rate of genetic gains per year in maize grain yield and key agronomic traits in pre-commercial and commercial varieties between 2008 and 2020 in Uganda. The genetic trends were dissected and profiled to quantify varietal selection and nomination into NPTs from NARO’s maize breeding program, CIMMYT, and private sector sources. We performed a multi-year analysis of historical, pre-commercial and commercial maize varieties to achieve these objectives. The historical study was compared with the replicated era studies conducted in 2015 and the country’s maize production trends between 1961 to 2020.

## 2 Materials and methods

### 2.1 NPT dataset

We used the data from maize NPTs conducted by NARO from 2008 through 2020. The trials were planted at eight locations, namely Abii, Bulengeni, Bulindi, Ikulwe, Masaka, Namulonge, Ngetta, and Serere, across the major maize-growing environments between 2008 and 2020 (**Table 1**). The NPTs were conducted in one or two seasons (seasons A and B) across the years. The number of entries in each trial ranged from 12 to 66, and the entries were allocated to sets one, two or three (1, 2, 3) based on the stage of submission to the NPT (**Table 2**). The experiments were laid out in an α-lattice design with two or three replications. Experiments were planted in two-row plots, in 5m long rows with 25cm interplant distance and 75 cm between rows, with a final plant density of approximately 53,333 plants ha^−1^. Agronomic management of the trials was carried out as recommended at each location. There was a total of 23-year by season by set combinations, referred to as trials. Each trial was conducted in four to seven locations (**Table 2**). An experiment was considered a year/season/test/location combination, resulting into 212 experiments (**Table 2**; **Supplementary Table A**). There were 419 entries of which 74 entries were from NARO’s mid-altitude maize breeding program, 211 from CIMMYT, 122 from private seed companies, and 12 commercial check entries in the analyzed trials (**Supplementary Table B**). Some commercial checks varied in the trials across the years but most were common in trials across years and provided connectivity to allow genetic gain estimates.

**Table 1:** Agro-metreological characteristics of the trial sites in Uganda.

Site	Longitude	Latitude	Altitude (masl)	Temperature		Mean annual rainfall 2008-2020 (mm)
				Min (°C)	Max (°C)	
Namulonge	32.63	0.52	1150	17	29	1270
Serere	33.44	1.54	1134	18	30	1239
Bulindi	31.44	1.50	1170	17	30	1176
Ngetta	32.92	2.29	1100	16	30	927
Bulegeni	34.32	1.30	1420	15	26	1400
Masaka	31.66	-0.30	1293	17	30	1190
Ikulwe	33.48	0.44	1209	17	29	1210
Abi	30.95	3.08	1208	19	35	1324

**Table 2:** Summary of the 23 maize National Performance Trials (NPTs) datasets in Uganda used for genetic gain estimation.

Year	NPT stage*	Season	No. of Locations	No. of Replications	No. of Entries	No. of Private Entries	No. of CIMMYT Entries	No. of NARO Entries	No. of Experiments
2008	1	A	6	2	23	4	0	19	6
2008	2	A	6	2	14	5	9	0	12
2009	1	B	5	2	13	7	0	6	5
2010	1	A	5	2	14	10	0	4	5
2011	1	B	6	2	23	12	1	10	6
2012	1	A	4	3	58	13	31	14	4
2012	2	B	4	2	32	3	15	14	8
2013	3	A	5	2	31	3	14	14	15
2013	3	B	6	2	23	1	9	13	18
2014	1	B	4	2	24	10	0	14	4
2015	1	B	7	2	35	6	29	0	7
2015	2	B	7	2	40	22	18	0	14
2015	3	B	7	2	51	17	29	5	21
2016	1	B	4	2	16	3	11	2	4
2016	2	B	2	2	12	2	9	1	4
2016	3	B	3	2	43	18	24	1	9
2017	1	A	4	2	66	21	34	11	4
2017	2	A	5	2	28	9	19	0	10
2017	3	A	5	2	37	13	24	0	15
2018	1	A	5	2	12	8	4	0	5
2018	3	A	5	2	61	23	38	0	15
2019	1	B	6	2	30	0	25	5	6
2020	3	A	5	3	23	13	10	0	15

*NPT Stage 1 is the first year and/or season testing following entry to NPT, Stage 2 is second season testing, and Stage 3 is the third season testing.

#### 2.3.1 Trait measurements

The entries were evaluated for grain yield and multiple agronomic traits (**Table 2**). All plants were hand-harvested, and grain weight was measured. Shelled grain weight was used to estimate grain yield corrected to 12.5% moisture content. Days to anthesis (AD) and silking were recorded when 50% of the plants had shed pollen, and 50% had silks, respectively. The anthesis–silking interval (ASI) was calculated as the difference between days to silking and days to anthesis. Plant height (PH) was measured in cm from the ground to the first tassel branch. Ear height (EH) was measured in cm as the distance from the ground to the insertion of the top ear. Ears per plant (EPP) was calculated as the number of ears harvested per plot divided by the number of plants per plot. Grain texture (GT) was recorded on a 1-5 scale where 1= more flint and 5 = very dent.

### 2.2 Era trials

The Era trial consisted of 54 maize varieties that were first available for testing in regional and on-farm trials from 1999 to 2017 (**Table 3**). The entries in this trial were 12 from NARO, 13 from CIMMYT, and 29 from the private sector. Of these, 39 varieties were released and are commercially produced. Of the 35 hybrid varieties released by the private sector, six were 100% CIMMYT/IITA-derived, 19 were combination hybrids derived using CGIAR’s elite maize lines and proprietary inbreds as parents, and 10 were completely based on germplasm from proprietary private sector. First-year testing in 1999 was represented by one entry, while 11 entries represented in 2015. The trial was conducted at six locations in the second (B) season of 2015. Each environment had two replications and each plot consisted of two 5-m long rows with 25-cm interplant distance and 75 cm between rows as above. Trial management, agronomic trait recording, measurement of grain weight, grain moisture, and estimation of grain yield were conducted as described for the NPT above.

**Table 3:** List of entries in the Era trial conducted at six locations in Uganda in 2015.

Entry	Name	Source	FYT	Entry	Name	Source	FYT
1	MM3	NARO	2009	28	SC721	SeedCo	2015
2	Longe 4	NARO	1999	29	SC727	SeedCo	2015
3	Longe 5D	NARO	2009	30	SC637	SeedCo	2015
4	Longe 6H	NARO	2000	31	SC641	SeedCo	2017
5	Longe 7H	NARO	2000	32	SC403	SeedCo	2015
6	Longe 8H	NARO	2000	33	SC301	SeedCo	2015
7	Longe 9H	NARO	2007	34	SC513	SeedCo	2015
8	Longe 10H	NARO	2012	35	SC529	SeedCo	2015
9	Longe 11H	NARO	2007	36	SC533	SeedCo	2015
10	UH5051	CIMMYT	2011	37	SC537	SeedCo	2015
11	UH5052	CIMMYT	2011	38	SC539	SeedCo	2015
12	UH5053	CIMMYT	2011	39	SC303	SeedCo	2015
13	UH5401	NARO	2008	40	WH505	Western Seed	2012
14	UH5402	NARO	2011	41	MRI-614	Syngenta	2014
15	UH5403	NARO	2011	42	MRI-624	Syngenta	2014
16	UH5354	CIMMYT	2012	43	MRI-634	Syngenta	2014
17	UH5355	CIMMYT	2012	44	MRI-724	Syngenta	2014
18	UH5556	CIMMYT	2012	45	MRI-734	Syngenta	2014
19	UH5557	CIMMYT	2014	46	PAN12	Pannar	2014
20	WE2114	CIMMYT	2012	47	PAN 7M-81	Pannar	2015
21	WE2115	CIMMYT	2012	48	PAN 7M-83	Pannar	2015
22	WE2101	CIMMYT	2012	49	PAN 8M-93	Pannar	2015
23	WE2103	CIMMYT	2012	50	KH500-43A	KALRO	2011
24	WE2104	CIMMYT	2012	51	DK8031	Bayer	2011
25	FS500S	FICA Seeds	2012	52	HYTECH 1100	Hytech Seed	2015
26	WE3106	CIMMYT	2014	53	HYTECH 2031	Hytech Seeds	2015
27	SC719	SeedCo	2015	54	EXPT HYRID 1	NASECO	2015

### 2.3 Uganda national maize average:

We obtained estimates of maize grain yield in Uganda from 1961 to 2020 from FAO statistics (https://www.fao.org/faostat/en/#data/QCL, verified March 10^th^, 2022). The data was categorized as follows: 1961 – 2020, 1999 – 2020 and 2008 – 2020 for analysis.

### 2.4 Statistical analysis

#### 2.4.1 NPT trial

An analysis of variance (ANOVA) was performed within each experiment using the model:


(1) $$y_{ij}=\mu +g_{i}+r_{j}+e_{ij}$$

where *y_ij_* is the mean phenotypic value of the trait within an experiment, *g_i_* is the random effect of the *i* ^th^ genotype with $g_{i}∼N(0,\sigma_{g_{i}}^{2})$ , *r_j_* is the random effect of the *j* ^th^ replication with $r_{j}∼N(0,\sigma_{r_{j}}^{2})$ , and *e_ij_* is the residual error with $e_{ij}∼N(0,\sigma_{e_{ij}}^{2})$ . Entry mean broad sense heritability (*H^2^*) within an experiment with *r* replications was calculated as


(2) $$H^{2}=\frac{\sigma_{g}^{2}}{\sigma_{g}^{2}+\left(\frac{\sigma_{e}^{2}}{r}\right)}$$

ANOVA was performed within each trial using the model:


(3) $$y_{ijk}=\mu +g_{i}+l_{j}+gl_{ij}+r{\left(l\right)}_{jk}+e_{ijk}$$

Where *y_ijk_* is the phenotype of the i^th^ genotype in the j^th^ location, in the k^th^ replication. *µ* is the overall mean; g_i_ is the random effect of the ith genotype with $g_{i}∼N(0,\sigma_{g_{i}}^{2})$ ; *l_j_* is the random effect of the j^th^ location with $l_{j}∼N(0,\sigma_{l_{j}}^{2})$ ; *r*(*l*)*_jk_* is a random effect of the k^th^ replication nested in the j^th^ location with $r{\left(l\right)}_{jk}∼N(0,\sigma_{r{\left(l\right)}_{jk}}^{2})$ ; *gl_ij_* is the interaction of the i^th^ genotype and j^th^ location with $gl_{ij}∼N(0,\sigma_{gl_{ij}}^{2})$ ; and *ϵ*_*ijk*_ is the error with $\epsilon_{ijk}∼N(0,\sigma_{\epsilon_{ijk}}^{2})$ . Entry mean heritability was calculated as:


(4) $$H^{2}=\frac{\sigma_{g}^{2}}{\sigma_{g}^{2}+\langle \frac{\sigma_{gl}^{2}}{l}\rangle +\langle \frac{\sigma_{e}^{2}}{lr}\rangle }$$

where l is the number of locations for that trial and r the number of replications. Only data from experiments with *H^2^* > 0.20 were used in the subsequent analysis.

Genetic gain in the NPT was estimated by regressing the estimated genetic value of each entry on the first year the entry was tested (FYT) in 2008 to 2020 data set. The FYT was the base year the entry was entered into the NPT. An experiment is a year/season/test/location combination, and an experiment is nested in a trial. We modeled the genotype x experiment interaction which encompasses genotype x location interaction. We declared a genetic gain to be significant if the probability of the slope was less than 0.05 and it was accompanied by an R^2^ value greater than 0.05. The genetic value of an entry was estimated using the mixed model:


(5) $$y_{ijklm}=\mu +g_{i}+c_{j}+t_{k}+x{\left(t\right)}_{kl}+r{\left(x\right)}_{lm}+gt_{ik}+gx{\left(t\right)}_{ikl}+\epsilon_{ijkl}$$

where *y_ijklm_* is the phenotypic value for genotype *i* tested in control group *j*, trial *k*, experiment l, and rep m. *µ* is the overall mean; *g_i_* is the fixed effect of the i^th^ genotype, *c_j_* is the fixed effect of the *j^th^* control group were the checks, where *j*=1 for the control population consisting of the two checks and *j*=2 for the tested group; *t_k_* is a random effect for the *k^th^* trial with $t_{k}∼N(0,\sigma_{t_{k}}^{2})$ ; *x*(*t*)*_kl_* is a random effect for the l*^th^* experiment nested in the k^th^ trial with $x{\left(t\right)}_{kl}∼N(0,\sigma_{x{\left(t\right)}_{kl}}^{2})$ ; *gt_ik_* is the random effect of the interaction of the i^th^ genotype and k^th^ trial with $gt_{ik}∼N(0,\sigma_{gt_{ik}}^{2})$ ; *gx(t)_ikl_* is the random effect of the interaction of the i^th^ genotype and l^th^ experiment nested in the k^th^ trial with $gx{\left(t\right)}_{ikl}∼N(0,\sigma_{gt_{ikl}}^{2})$ ; *r*(*x*)*_lm_* is a random effect of the m^th^ replication nested in the l^th^ experiment with $r{\left(x\right)}_{lm}∼N(0,\sigma_{r{\left(x\right)}_{lm}}^{2})$ ; and *ϵ*_*ijklm*_ is the residual error with $\epsilon_{ijklm}∼N(0,\sigma_{\epsilon_{ijklm}}^{2})$ . Correlation analysis of control means and entry means was carried to assess the efficiency of using controls to substitute for experiment effect.

The model in equation 5 was run separately for all entries: only entries from private sources, only entries from CIMMYT sources, and only entries from NARO. The same controls were used in each analysis. The Best Linear Unbiased Estimator (BLUE) for each entry from equation 5 was then regressed onto the FYT value for that entry. Data from the checks were excluded from the regression analysis. The regression was done separately for each source of entries. The slope of this regression was used as the estimate of genetic gain. The percent genetic gain per FYT was calculated as the (slope/*u*) *100, where u is the intercept in equation 5. For GY, the analysis was run across trials, and separately for trials with mean GY > 3.3 t/ha (high), and for trials with mean GY< 3.3 t/ha (low). We assessed the significance of the source of the NPT entries using the model shown in equation 5 by adding a term for the n^th^ source (*s_n_*) as a fixed effect and changing *g*_i_ to be the effect of the i^th^ genotype nested in the n^th^ source, *g(s)_in_*.

#### 2.4.2 Era trial

Broad-sense heritability was calculated over all environments, as shown in equation 4 above.

The first-year testing (FYT) effect was assessed in two ways. First, we analyzed variance using FYT as a covariate using the model:


(6) $$y_{ijkl}=\mu +f_{i}+g_{j}+v_{k}+r{\left(v\right)}_{kl}+gv_{jk}+\epsilon_{ijkl}$$

where *y_ijkl_* is the phenotypic value for genotype *j*, from FYT i, tested in environment *k*, and rep *l. µ* is the overall mean; *f_i_* is the fixed effect of the i^th^ FYT, *g_j_* is the fixed effect of the j^th^ genotype, *v_k_* is the random effect of the k*^th^* environment with $v_{k}∼N(0,\sigma_{v_{k}}^{2})$ ; *gv_jk_* is the random effect of the interaction of the j^th^ genotype and k^th^ environment with $gv_{jk}∼N(0,\sigma_{gv_{jk}}^{2})$ ; *r*(*v*)*_kl_* is a random effect of the l^th^ replication nested in the k^th^ environment with $r{\left(v\right)}_{kl}∼N(0,\sigma_{r{\left(v\right)}_{kl}}^{2})$ ; and *ϵ*_*ijkl*_ is the residual error with $\epsilon_{ijkl}∼N(0,\sigma_{\epsilon_{ijkl}}^{2})$ . The model was run once with *fi* to test its significance and then once without *fi* to obtain BLUEs for each entry. These BLUEs were then regressed onto their FYT values to estimate the effect of increasing FYT. A genetic gain was declared significant if the FYT covariate test had a probability of P< 0.05, and the slope was significant at P< 0.05.

#### 2.4.3 Uganda national average maize yield, 1961 - 2020

Analysis of the was done across all 60 years of average maize yield estimates, for 22 years (1999–2020) to reflect the range of years of entries in the Era trial, and for 13 years (2008–2020) to reflect the range of years for entries in the NPT. The national average maize yield was regressed over the baseline years.

## 3 Results

### 3.1 National performance trials

There was considerable variation among the experiment means and heritability for the traits examined in this study (**Table 3**). GY, AD, PH and EH showed moderate heritability (0.40–0.59), while EPP and ASI had low heritability (<0.32) within experiments. Only GT could be considered highly heritable with an average heritability of 0.67. Entry mean heritability estimated over all experiments within a trial (year/test/season combination) was greater than heritability within experiments. Heritability within a trial showed similar trends of high, moderate, and low heritability for traits as within experiment estimates (**Table 4**).

**Table 4:** List of traits assessed, and a summary of the average trait values and entry mean heritability within an experiment.

Code	Description		Experiment Trait Value	Heritability within an Experiment			Heritability within a Trial		
		No. of Exp.	Mean	Mean	% > 0.5	%< 0.2	Mean	% > 0.5	%< 0.2
GY	Grain yield (T/ha)	116	4.2	0.49	54.3	19	0.63	0.78	0.13
AD	Day to Anthesis	107	66.7	0.59	55.1	11.2	0.62	0.73	0.14
ASI	Anthesis-silking interval	98	1.6	0.32	22.4	35.5	0.27	0.23	0.55
PH	Plant height (cm)	83	193.1	0.4	32.5	24.1	0.64	0.82	0.09
EH	Ear height (cm)	84	92.3	0.42	36.5	21.2	0.65	0.77	0.09
EPP	Ears per plant	99	1	0.31	25.3	40.4	0.26	0.22	0.43
GT	Grain texture (1-5)	115	2.5	0.67	82.6	7	0.8	0.91	0

An experiment was defined as a year/test/season/location combination.

We obtained BLUEs using a model that used the checks as a fixed effect to adjust for the effect of experiments. Correlation analysis of the entry means with control/check means within experiments and within trials indicated that control means were a suitable surrogate for experiment effect for most traits with correlations greater than 0.70 for 5 of the 7 traits (**Table 5**). The control means were poorly correlated to the mean of all entries for AD and ASI.

**Table 5:** Correlation of entry means with control (check) means by NPT experiment, trial, and year.

Trait	Correlation of control means and entry means in the NPT		
	By Experiment	By Trial	By Year
Grain yield	0.88	0.83	0.81
Days to anthesis	0.48	0.23	-0.03
Anthesis-silking interval	0.44	0.49	0.82
Plant height	0.93	0.79	0.77
Ear height	0.90	0.76	0.88
Ears per plant	0.83	0.62	0.09
Grain texture	0.73	0.73	0.81

#### 3.1.1 Grain yield

**Figure 1:**
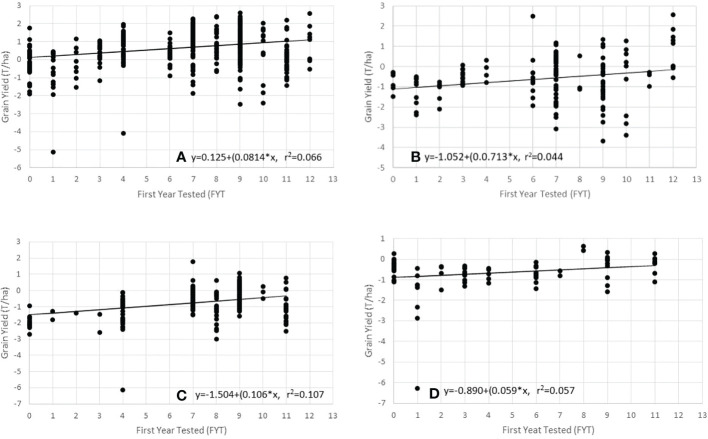
Regression of grain yield (GY) on to the First Year of Testing (FYT) using data from the Ugandan NPT trials. **(A)** all entries, **(B)** entries from private sector, **(C)** entries from CIMMYT sources, and **(D)** entries from NARO.

There was a significant difference (*P*< 0.05) in genetic gain for hybrid yields from all sources entered into the NPT (**Table 6**). Evidence of significant genetic gain for increasing GY over all experiments was noted in all entries and among entries from each of the three sources (**Table 6**; **Figure 1**). When analysis was done by source, entries from CIMMYT showed the higher GY mean (5.37 t/ha) and genetic gain estimate (1.98% year^-1^ or 106 kg ha^-1^ year^-1^). Entries from NARO and private sector sources had lower mean GY of 4.56 t ha^-1^ and 4.62 t ha^-1^, respectively. Entries from NARO had the lowest estimated genetic gain (1.30% year^-1^ or 59 kg ha^-1^ year^-1^).

**Table 6:** Results from regression of the Best Linear Unbiased Estimators (BLUEs) for grain yield and other agronomic traits of entries in the National Performance Trials (NPTs) by source onto their first year of testing (FYT).

Trait †	Source	N	FYT Slope	Intercept	Prob	R2	Mean	Slope as % mean
GY	All	419	0.081	0.127	<0.001	0.066	3.62	2.25
All	Private	122	0.079	-1.110	0.015	0.051	4.62^b^	1.71
Envs	CIMMYT	211	0.106	-1.506	<0.001	0.107	5.37^a^	1.98
	NARO	74	0.059	-0.890	0.027	0.057	4.56^b^	1.30
GY	All	419	0.067	0.157	0.003	0.024	4.33	1.55
High	Private	122	0.101	-1.290	0.010	0.056	5.20^b^	1.94
Envs	CIMMYT	211	0.072	-1.200	<0.001	0.030	5.92^a^	1.22
	NARO	74	0.135	-1.570	0.004	0.148	5.15^b^	2.62
GY	All	419	0.004	0.557	0.834	0.000	1.55	0.26
Low	Private	122	-0.032	0.655	0.422	0.008	1.42^b^	-2.25
Envs	CIMMYT	211	-0.094	1.300	0.001	0.073	1.79^a^	-5.25
	NARO	74	0.010	1.870	0.087	0.004	1.83^a^	0.55
AD	All	334	-0.215	-1.571	<0.001	0.075	69.08	-0.31
	Private	122	-0.506	5.540	<0.001	0.235	63.96^a^	-0.79
	CIMMYT	186	-0.353	2.816	<0.001	0.074	65.31^b^	-0.54
	NARO	69	-0.125	4.652	0.086	0.043	63.22^a^	-0.2
ASI	All	331	0.019	-1.267	0.45	0.002	2.86	0.67
	Private	122	0.049	-1.305	0.063	0.029	2.94^a^	1.66
	CIMMYT	186	0.052	-4.781	0.228	0.008	1.62^a^	3.21
	NARO	69	-0.022	-0.55	0.91	0	2.00^a^	-1.1
PH	All	332	0.643	-12.4	0.048	0.012	202.3	0.32
	Private	122	0.92	-20.7	0.021	0.043	210.50^a^	0.44
	CIMMYT	186	0.857	1.69	0.022	0.028	185.90^b^	0.46
	NARO	69	1.582	-0.46	0.001	0.155	184.70^b^	0.86
EH	All	326	-0.342	-9.98	0.076	0.01	104.3	-0.33
	Private	122	-0.156	-16.89	0.652	0.002	111.40^a^	-0.14
	CIMMYT	186	-0.496	6.31	0.074	0.017	86.61^b^	-0.57
	NARO	69	0.801	1.56	0.015	0.085	86.30^b^	0.93
EPP	All	376	0.002	-0.013	0.264	0.003	0.96	0.22
	Private	121	-0.002	-0.017	0.547	0.003	0.99^a^	-0.15
	CIMMYT	211	-0.01	0.075	<0.001	0.091	0.99^a^	-1.05
	NARO	74	-0.007	0.053	0.028	0.065	0.97^a^	-0.72
GT	All	407	0.007	-0.085	0.488	0.001	2.64	0.27
	Private	122	0.022	0.003	0.188	0.014	2.54^b^	0.85
	CIMMYT	211	-0.078	0.482	<0.001	0.077	2.75^a^	-2.84
	NARO	74	0.069	0.483	0.001	0.147	2.64^b^	2.6

^†^GY, grain yield; AD, anthesis date; ASI, anthesis to silking interval; PH, plant height; EH, ear height; EPP, ear placement position; GT, grain texture.

^a,b,c^ indicate trait means that are significantly different based on Duncan’s multiple range test. “N” indicates the number of entries in each analysis.

We also analyzed across the different sources of germplasm for high GY experiments (mean GY >3.3 t ha^-1^) and low GY experiments (mean GY< 3.3 t ha^-1^). The overall genetic gains in the high mean GY experiments were lower compared to that estimated across all environments. The genetic gain estimates for high mean GY experiments varied by source relative to estimates from all experiments with some increasing and others reducing (**Table 6**). The estimated genetic gain for the NARO and private sector entries were greater in the high GY experiments than in overall experiments. The genetic gains for CIMMYT hybrids were found to be lower in the high yielding environments (72 kg ha^-1^ year^-1^) compared to 106 kg ha^-1^ year^-1^ across all environments. The genetic gains were lower in the low GY experiments for the different germplasm sources (**Table 6**). Only the slope from CIMMYT analysis was significant and indicated that the GY had decreased over time (-94 kg ha^-1^ year^-1^) in lower mean GY experiments.

#### 3.1.2 Other agronomic traits

There was significant (*P*< 0.05) genetic gain for decreasing days to anthesis when analyzed over all entry sources or within just the CIMMYT and commercial entries (**Table 6**). The gain was highest for entries from the private sources, with a decrease of AD of -0.79% year^-1^. This regression produced the greatest r^2^ value (0.235) of any trait/source combination. Reduced days to anthesis was observed in all the entries though the change could not always be declared significant. Entries from NARO had a lower AD mean compared with CIMMYT entries, which had the highest AD mean among all sources. There was evidence of increasing PH in the entries in the NPT though the only significant gain was for the NARO entries, where the increase was 1.58 cm year^-1^, or a gain of 0.89% year^-1^ (**Table 6**). The CIMMYT and NARO entries were generally shorter than the entries from the private sector. Similar to PH, the only significant increase for EH was among the NARO entries, where EH increased by 0.80 cm year^-1^ at a rate of 0.93% year^-1^ (**Table 6**). EH was significantly greater in the private sector entries than in the CIMMYT or NARO entries. Significant (*P*< 0.05) genetic gain for EPP was noted among the CIMMYT and NARO entries, where the decrease was -1.05% and -0.72% year^-1^, respectively (**Table 6**). Changes in EPP were negligible for the private sector entries. There were no significant differences for EPP among the sources of entries. Significant genetic gain was noted for GT among the CIMMYT and NARO entries (**Table 5**). GT decreased among the CIMMYT entries at -2.84% year^-1^, while it increased among the NARO entries at a rate of 2.60% year^-1^. The CIMMYT entries had the higher average GT value.

### 3.2 Era trial

Broad-sense heritability ranged from 0.50 to 0.96 for the key traits (**Table 7**). Mean GY was 3.3 t ha^-1^ and heritability was 0.79. Genetic gain in the era trial was assessed by testing the significance of FYT as a covariate and by a regression analysis similar to that of the NPT trials. The test had 54 entries with FYT values ranging from 1999 to 2017 (**Table 2**).

**Table 7:** Means from each environment for all traits assessed in the era trial, along with heritability overall traits.

Environment	GY †	AD	SD	ASI	PH	EH	EPP	GT
BULINDI	4.6	67.7	66.4	2.1	.	.	.	2.92
IKULWE	4.1	63.9	64.9	1.1	239	117	0.9	2.62
Masaka	2.3	66.5	.	3.4	185	82	1.1	1.61
NAMERD	3.4	67.8	.	1.1	214	114	1.2	2.60
NGETTA	1.1	62.0	66.8	4.9	198	113	.	1.83
SERERE	4.0	64.2	65.6	1.4	210	103	.	2.61
No. of Environs	6	6	4	6	5	5	3	6
Mean	3.3	65.4	65.9	2.3	209.2	105.8	1.1	2.4
H^2^	0.79	0.96	0.23	0.50	0.74	0.81	0.10	0.92

^†^GY, grain yield; AD, anthesis date; ASI, anthesis to silking interval; PH, plant height; EH, ear height; EPP, ear placement position; GT, grain texture. The dots (·) in the table denote missing values.

The analysis of GY was performed over all the six environments, the four highest yielding environments (GY > 3.3 t ha^-1^) and the two lowest yielding sites (**Table 7**). Significant genetic gain for GY was obtained in all three analyses. The largest estimated genetic gain was found in the high yielding environments at a rate of 61 kg ha^-1^ year^-1^, though this gain was the lowest on a percentage basis (1.52% year^-1^) (**Table 8**). The genetic gain estimate was lowest in the low yielding environments (43 kg ha^-1^ year^-1^) although this gain was greatest on a percentage basis (2.52% year^-1^). The annual gains (43 to 61 kg ha^-1^ year^-1^) in the era trial were lower than those estimated in the NPT trial (59 to 81 kg ha^-1^ year^-1^) (**Table 6**).

**Table 8:** Results from ANOVA and regression analyses of the era trial conducted at six locations in 2015.

		ANOVA		Regression				
Trait†	Environments	FYT Prob	Mean	Slope	Intercept	Prob	r^2^	Slope as % mean
GY	All	<0.001	3.25	0.055	-109.23	0.037	0.081	1.69
GY	High Yield	<0.001	4.02	0.061	-121.81	0.047	0.065	1.52
GY	Low Yield	<0.001	1.70	0.043	-85.10	0.012	0.118	2.52
AD	All	0.31	65.3	-0.023	46.87	0.788	0.001	-0.04
ASI	All	0.88	2.17	0.005	-9.18	0.876	0.001	0.21
PH	All	0.04	194.7	0.394	-778.00	0.296	0.021	0.20
EH	All	0.34	101.3	0.138	-273.00	0.669	0.004	0.14
EPP	All	0.91	1.01	0.002	-2.08	0.917	0.000	0.15
GT	All	0.00	3.21	0.022	-45.86	0.184	0.034	0.70

^†^GY, grain yield; AD, anthesis date; ASI, anthesis to silking interval; PH, plant height; EH, ear height; EPP, ear placement position; GT, grain texture.

### 3.3 Uganda national data

**Figure 2:**
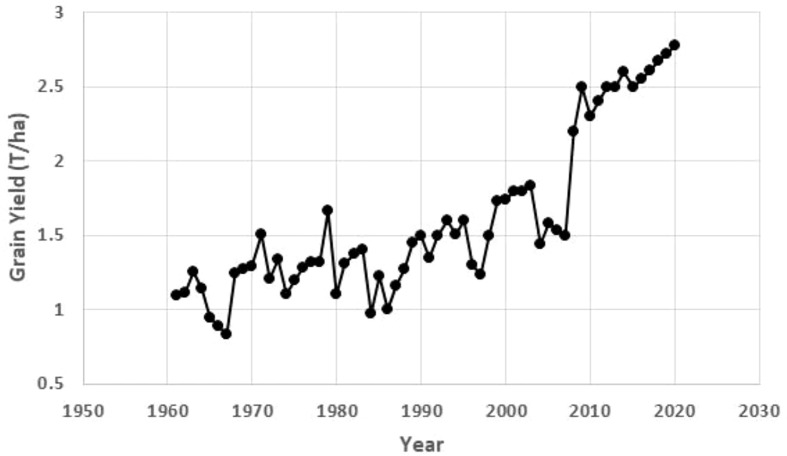
Annual grain yield of maize in Uganda using data from FAO (https://www.fao.org/faostat/en/#data/QCL).

All three regressions of grain yield onto year were significant (*P<*0.0001), accounting for more than 72% of the variation (**Table 9**; **Figure 2**). The greatest gain per year was in the 1999-2020 period at 62 kg ha^-1^ year ^-1^ (2.85% per year).

**Table 9:** Regression of estimated national average maize grain yields in Uganda, onto a baseline year over three periods.

Period	Slope (t/ha/year)	Intercept	Prob	r^2^	Mean (t ha^-1^ year^-1^)	Slope as % mean
1961-2020	0.026	-50.9	<0.0001	0.729	1.603	1.65
1999-2020	0.062	-122.4	<0.0001	0.766	2.172	2.85
2008-2020	0.037	-72.7	<0.0001	0.815	2.525	1.48

## 4 Discussion

This study estimated genetic gains in public and private maize breeding programs using data from the national performance trials and the era trials, besides the trends in Ugandan national average maize yields. Each of these datasets had its limitations; the NPT had an unbalanced dataset where the checks were used as surrogates to estimate and adjust for experimental effect. The use of controls was effective for most of the traits. The advantage of the NPT over the era trial was that there were many entries within FYT category. The era trial was balanced with all entries tested in all the six environments but had fewer entries per FYT group as compared to the NPT. The entries in the NPT are for varietal release to commercialization from the most elite end-products of selections from breeding pipelines. Selection of hybrids for entry into the NPTs was made from advanced yield trials of diverse breeding pipelines. Neither the NPT nor the era trial could be considered as an evaluation of genetic gain in a specific breeding pipeline. Nevertheless, analysis of NPT and era trials can shed light on the effectiveness of breeding programs in delivering genetic gains to the farmers. Several studies have estimated genetic trends in maize and other crops using variety trials (Mackay et al., 2011; Barrero Farfan et al., 2013; Laidig et al., 2014) rather than era trials which are more expensive to conduct.

In this study, the overall genetic gain estimated from NPT was 81 kg ha^-1^ year^-1^. This genetic gain estimate was lower than the 109 kg ha^-1^ yr^-1^ reported for CIMMYT’s maize hybrid breeding pipeline (Masuka et al., 2017a) but comparable to the 79 kg ha^-1^ yr^-1^ for CIMMYT’s OPV breeding pipeline (Masuka et al., 2017b) in Eastern and Southern Africa (ESA). These genetic gains are attributed to the use of higher yielding and stress-tolerant parental inbred lines in ESA. Recent reports indicated that there is improvement in the grain yield performance (1.4% year^-1^ or 39 kg ha^-1^ yr^-1^) of the inbred lines that are used to develop new products that are allocated and released for commercial production (Worku et al., 2020). This genetic improvement is reflected in the recent analysis of genetic trends in CIMMYT’s breeding pipeline in ESA of up to 138 kg ha^-1^ yr^-1^ (Prasanna et al., 2022b). In other studies, genetic gains of 40 kg ha^-1^ year^-1^ have been reported in West Africa (Badu-Apraku et al., 2015). Edmeades (2013) reported genetic gains of 39 to 80 kg ha^-1^ year^-1^ in sub-Sahara Africa under well-watered conditions but just 18 kg ha^-1^ year^-1^ under drought stress. In other regions, genetic gains of 65 to 75 kg ha^-1^ year^-1^ in the USA (Duvick, 2005), and 74 and 131 kg ha^-1^ year^-1^ in China (Ci et al., 2011; Ma et al., 2015), were reported.

The estimated genetic gains under high yielding environments were similar between the NPT (1.55%) and the era trials (1.52%). This shows that either of these datasets can be used to obtain reliable estimates of genetic gain, but NPTs offer a cheaper option for the estimates. The high overall genetic gain (2.25%) in NPTs obtained from all collaborators indicates that a combined effort of public-private partnerships is critical in delivering improved genetics to the farmers. The results showed that CIMMYT hybrids were significantly higher yielding than the NARO and private sector entries in the NPTs. This highlights the need for continuous improvement in breeding, for integrating new tools and methods to drive rates of genetic gain with sustainable funding for the public sector. We also noted significantly lower rates of genetic gain in the low GY experiments.

In broad terms, currently grown maize varieties in Uganda have higher GY, have an earlier anthesis date, and are taller than those cultivated in the past, as these traits showed the same trend in each source of NPT entries and in the era trials (**Tables 6**–**8**). The traits with the strongest evidence of genetic gains were GY, AD and GT. All five traits had an average within-trial heritability greater than 0.40 in the NPT, with GY, AD, and GT having average heritability greater than 0.60. All breeders seem to select more intensely for GY and AD. Grain yield and AD as a proxy for early maturity are important farmer-preferred traits (Worku et al., 2020).

The genetic trends indicated that grain texture significantly decreased over time in the CIMMYT entries tending to be more flint-type, but we observed a significant increase in the grain texture of the NARO entries, towards selection for dent type. The trends for PH and EH were interesting because while PH increased in all sources, EH was unchanged in the CIMMYT and private sector sources but increased in the NARO entries. Increasing PH over time probably indicates selection for more vigorous plant types. With product profile-based breeding and foreseen mechanized operations among emerging commercial farmers, there is a need to breed and select for shorter and more modern plant types for increased plant population density and higher harvest index.

Maize productivity in Uganda increased for the three truncated periods analyzed (1961 – 2020, 1999 – 2020 and 2008 – 2020). This significant upward trend of the national average maize yields in Uganda could be partly due to improved genetics and production practices. The highest national maize productivity was achieved between 1999 and 2020, consistent with a systematic and sustained maize breeding program that resulted in the release of the first suite of improved stress-tolerant hybrids in 1999. The first formal release of maize varieties in Uganda was in 1960 followed by another release of Kawanda Composite A in 1971. These varieties were grown for a long time until the late 1980s when they succumbed to the maize streak virus (MSV) during the outbreak of the disease in 1987. These two open-pollinated varieties (OPVs) were lost due to a combination of lack of maintenance breeding and civil strife between 1980 and 1986. The varieties developed later took into account breeding for host plant resistance for MSV and other major foliar diseases like Turcicum leaf blight (caused by *Exserohilum turcicum* (Pass.) K. J. Leonard & Suggs) and gray leaf spot (caused by *Cercospora zeae-maydis* Tehon & E. Y. Daniels). This genetic improvement program of maize in Uganda, supported initially by USAID and later by the Rockefeller Foundation, partly explains the highest national productivity gains (62 kg ha^-1^ year^-1^) starting from 1999. The other major contributing factor was the advent of the private seed sector that took over from government managed seed industry in the mid-1990s.

Analysis of the national productivity trends showed lower genetic gains in the increase of the mean yield (37 kg ha^-1^ year^-1^) for 2008 – 2020, compared to 59 to 106 kg ha^-1^ year^-1^ (**Table 6**) for the same period of NPT. This indicates that improved genetics is most likely contributing to the increased maize yield in Uganda but farmers may not be reaping the entire benefit of improved stress-tolerant varieties due to multiple reasons. These include: 1) not quickly adopting the improved varieties; 2) using low yield environments for maize production; and 3) use of poor agronomic practices which limit the expression of the improved genetics. Given the success of breeding for improved yield and stress tolerance, these issues need to be addressed to reduce the yield gaps. Poor varietal turnover is common in East Africa and remains a major bottleneck in improving crop yields (Abate et al., 2017; Atlin et al., 2017; Lee, 2020; Chivasa et al., 2022). Varietal turnover appears to be better in Uganda compared to other East African countries; the area-weighted average age of maize varieties in Uganda is about 7.7 years compared to an average age of 10.2 years across ESA (Chivasa et al., 2022). Improved agronomic practices including timely planting, fertilizer application, and weed management have the potential to further improve maize productivity in Uganda, together with improved genetics.

## 5 Conclusions

This study estimated the genetic gains for grain yield and some key agronomic traits in pre-commercial and commercial maize varieties from different breeding programs (NARO, CIMMYT and private sector) using datasets from the NPTs (2008 – 2020) and the era trials on varieties released between 1999 – 2020 in Uganda. The results revealed significant annual genetic gains for grain yield and agronomic traits over the years. The study indicates that breeding for the target maize-growing environments in Uganda has been successful, with significant contribution to maize improvement from the private sector, CIMMYT and NARO supported through increased collaboration and expanded testing networks. The rates of gains estimated from the national maize yield average were lower than those obtained from other estimates but these would be higher if farmers use appropriate crop management practices, including the use of fertilizers, which is still very low in Uganda (less than 6 kg/ha on average) (Rware et al., 2020). The sustained increase in maize production in the country could be attributed to a combination of various factors but primarily the infusion of improved stress-tolerant maize varieties and replacement of obsolete varieties due to increasing investment by the private sector, coupled with production by both small-scale and emerging large-scale farmers (Chivasa et al., 2022). Farmers in Uganda have switched to mainly growing hybrids that yield better than OPVs, most of which have been withdrawn and discontinued (Chivasa et al., 2022).

The finding from this study demonstrated continuous improvement in terms of realized genetic gains and productivity gains under research and on-farm conditions, respectively. To achieve larger genetic gains there is a need to modernize the breeding programs by incorporating use of molecular markers and doubled haploid technologies, faster recycling of elite lines, and switch to product profile-based breeding with optimized breeding schemes to improve selection efficiency. In the last three years, NARO’s breeding program has been assessed, and improvement plans and investment cases have been identified under the CGIAR Excellence in Breeding (EiB) platform and Accelerating Genetic Gains for Maize and Wheat Improvement (AGG) Project. These new breeding strategies now inform the product-profile based variety development and replacement. Gaps in seed systems still need to be addressed to effectively deliver improved stress-resilient varieties to the smallholder farmers for greater impact.

## Data availability statement

The raw data supporting the conclusions of this article will be made available by the authors, without undue reservation.

## Author contributions

GA, DK, and CK – conceptualization, field experimentation, data analysis and manuscript development. CS – conceptualization, data analysis and manuscript development and review. BD and LM – conceptualization, manuscript review and funding. DM and YB – germplasm development, experimentation and manuscript review and, BP – manuscript review, editing, and funding. All authors contributed to the article and approved the submitted version.
