# Supplementary material for: Genetic trends for yield and key agronomic traits in pre-commercial and commercial maize varieties between 2008 and 2020 in Uganda
Source: Front Plant Sci. 2023 Mar 10;14:1020667. doi: 10.3389/fpls.2023.1020667 (PMC10036907; doi:10.3389/fpls.2023.1020667)
Supplement: Supplementary file 1 [file DataSheet_1.docx]

Supplemental Table A. List of all experiments (year/NPT/season/location combination) in the 2008-2020 National Performance Trial conducted by the National Agriculture Research Organization of Uganda. The trait means for each experiment is presented: GY=grain yield; AD=days to anthesis; ASI=interval between anthesis and silking; PH-plant height; EH=ear height; EPP=ears per plant; GT=grain texture.

|  |  |  |  | GY | AD | ASI | PH | EH | EPP | GT |
| --- | --- | --- | --- | --- | --- | --- | --- | --- | --- | --- |
| Year | NPT | Season | Location | T/ha | Days | days | cm | cm | count | 1-5 |
| 2008 | 1 | A | Bulegeni | 6.30 | . | . | . | . | 0.98 | 2.90 |
| 2008 | 1 | A | Bulindi | 3.13 | 71.35 | 3.42 | 151.83 | 70.35 | 0.70 | 2.07 |
| 2008 | 1 | A | Masaka | 1.14 | 79.09 | 4.91 | . | . | 0.54 | 1.96 |
| 2008 | 1 | A | Namulonge | 4.20 | 65.40 | 2.85 | 147.58 | 71.33 | 0.96 | 1.86 |
| 2008 | 1 | A | Ngetta | 6.44 | 58.77 | 2.96 | 218.77 | 114.56 | 1.14 | 1.93 |
| 2008 | 1 | A | Serere | 5.22 | 67.13 | 0.38 | 201.25 | 90.10 | 2.07 | 2.02 |
| 2008 | 2 | A | Bulegeni | 8.28 | . | . | . | . | 0.96 | 3.45 |
| 2008 | 2 | A | Bulindi | 3.31 | 73.13 | 4.67 | 144.83 | 63.50 | 0.93 | 2.07 |
| 2008 | 2 | A | Masaka | 1.08 | 84.53 | -1.50 | . | . | 0.58 | 2.00 |
| 2008 | 2 | A | Namulonge | 4.84 | 62.83 | 3.67 | 176.17 | 95.70 | 0.96 | 2.28 |
| 2008 | 2 | A | Ngetta | 6.05 | 57.62 | 2.38 | 223.30 | 123.20 | . | 2.10 |
| 2008 | 2 | A | Serere | 4.06 | 66.73 | 0.80 | 185.17 | 82.57 | 1.06 | 2.67 |
| 2009 | 1 | B | Bulindi | 3.42 | 66.25 | 1.19 | 168.13 | 76.50 | 0.97 | 2.44 |
| 2009 | 1 | B | Masaka | 7.86 | 70.84 | 1.72 | 233.38 | 114.19 | 1.15 | 2.22 |
| 2009 | 1 | B | Namulonge | 5.21 | 62.81 | 0.94 | 204.19 | 97.68 | 1.06 | 2.13 |
| 2009 | 1 | B | Ngetta | 2.73 | 60.10 | 6.27 | 133.73 | 52.93 | 0.91 | 1.83 |
| 2009 | 1 | B | Serere | 6.38 | 62.10 | 0.55 | 199.81 | 96.97 | 1.12 | 2.57 |
| 2010 | 1 | A | Bulindi | 5.75 | 63.50 | 0.97 | 195.92 | 103.78 | 1.09 | 2.28 |
| 2010 | 1 | A | Masaka | 3.87 | 69.47 | 2.03 | 168.31 | 80.28 | 0.92 | 2.61 |
| 2010 | 1 | A | Namulonge | 3.47 | 60.28 | 2.03 | 193.86 | 92.81 | 0.99 | 1.92 |
| 2010 | 1 | A | Ngetta | 8.13 | 65.92 | 1.69 | 237.78 | 121.17 | . | 3.24 |
| 2010 | 1 | A | Serere | 5.21 | 67.58 | 1.61 | 223.47 | 105.00 | 1.00 | 3.13 |
| 2011 | 1 | B | Bulegeni | 1.83 | 79.06 | 2.65 | 171.32 | 80.11 | 0.69 | 2.44 |
| 2011 | 1 | B | Bulindi | 3.50 | 65.26 | 0.87 | 187.69 | 97.85 | 1.09 | 2.62 |
| 2011 | 1 | B | Masaka | 2.26 | 76.46 | 2.43 | 159.69 | 66.65 | 0.90 | 2.36 |
| 2011 | 1 | B | Namulonge | 4.07 | 66.22 | 1.20 | 202.50 | 98.19 | 1.01 | 2.40 |
| 2011 | 1 | B | Ngetta | 2.46 | 60.85 | 3.02 | 221.70 | 99.11 | 1.04 | 2.22 |
| 2011 | 1 | B | Serere | 3.23 | 82.70 | -0.43 | 250.56 | 136.06 | 1.01 | 2.46 |
| 2012 | 1 | A | Bulindi | 6.27 | 64.38 | 1.06 | 224.53 | 110.56 | 0.92 | 2.61 |
| 2012 | 1 | A | Namulonge | 5.78 | 65.13 | 0.89 | 214.04 | 107.58 | 0.88 | 2.43 |
| 2012 | 1 | A | Ngetta | 8.16 | 59.79 | 1.26 | 218.19 | 107.70 | 0.94 | 2.61 |
| 2012 | 1 | A | Serere | 8.10 | 62.34 | -2.80 | 239.27 | 126.17 | 1.00 | 2.78 |
| 2012 | 2 | B | Masaka | 5.30 | 69.16 | -1.77 | . | . | 0.99 | 2.30 |
| 2012 | 2 | B | Namulonge | 5.85 | 64.23 | -0.09 | 212.33 | 94.55 | 1.17 | 2.19 |
| 2012 | 2 | B | Ngetta | 0.82 | 68.07 | -1.85 | 178.87 | 91.16 | 1.00 | 2.74 |
| 2012 | 2 | B | Serere | 4.40 | 67.60 | 4.46 | 194.42 | 114.88 | 1.06 | 3.24 |
| 2013 | 3 | A | Bulegeni | 5.39 | 69.04 | 0.79 | 202.07 | 88.54 | 0.96 | 2.78 |
| 2013 | 3 | A | Bulindi | 4.57 | 66.65 | 1.93 | . | . | 1.05 | 2.21 |
| 2013 | 3 | A | Namulonge | 1.36 | 67.31 | 6.55 | 115.62 | 59.93 | 1.07 | 1.90 |
| 2013 | 3 | A | Ngetta | 6.32 | 62.00 | 2.72 | . | . | 1.03 | 2.49 |
| 2013 | 3 | A | Serere | 3.27 | 64.64 | 3.28 | 171.63 | 74.88 | 1.07 | 2.54 |
| 2013 | 3 | B | Bulindi | 1.76 | 71.21 | 1.37 | 127.78 | 57.33 | 1.03 | 1.95 |
| 2013 | 3 | B | Ikulwe | 1.44 | 65.56 | 1.13 | . | . | . | 2.49 |
| 2013 | 3 | B | Masaka | 4.39 | 71.06 | 1.85 | 220.40 | 107.14 | 1.27 | 2.74 |
| 2013 | 3 | B | Namulonge | 3.39 | 64.60 | 2.06 | 166.04 | 88.07 | 1.05 | 1.53 |
| 2013 | 3 | B | Ngetta | 2.15 | . | . | 157.18 | 73.14 | 0.98 | 2.22 |
| 2013 | 3 | B | Serere | 2.98 | 62.02 | 0.98 | 178.64 | 82.12 | 1.10 | 2.16 |
| 2014 | 1 | B | Ikulwe | 3.85 | 63.70 | 1.84 | 202.80 | 99.00 | 0.91 | 2.45 |
| 2014 | 1 | B | Namulonge | 1.97 | 73.68 | 1.50 | 176.75 | 90.80 | 0.98 | 3.11 |
| 2014 | 1 | B | Ngetta | 0.89 | 65.30 | 2.91 | 197.91 | 107.25 | . | 2.02 |
| 2014 | 1 | B | Serere | 2.07 | 67.09 | 2.41 | 187.75 | 90.88 | . | 2.88 |
| 2015 | 1 | B | Bulegeni | 2.47 | 74.50 | 3.58 | 157.14 | 62.93 | 1.00 | 3.40 |
| 2015 | 1 | B | Bulindi | 5.82 | 67.89 | 1.13 | . | . | 1.00 | 2.66 |
| 2015 | 1 | B | Ikulwe | 3.94 | 62.56 | 0.44 | 223.35 | 108.01 | 0.90 | 2.82 |
| 2015 | 1 | B | Masaka | 5.40 | 70.26 | 1.73 | . | . | 0.91 | 2.06 |
| 2015 | 1 | B | Namulonge | 4.69 | 65.56 | 0.28 | 211.27 | 94.41 | 1.10 | 2.49 |
| 2015 | 1 | B | Ngetta | 1.35 | 62.30 | 2.78 | 156.44 | 76.25 | 1.00 | 1.76 |
| 2015 | 1 | B | Serere | 3.84 | 63.55 | 0.60 | 181.45 | 85.66 | 0.92 | 2.51 |
| 2015 | 2 | B | Bulegeni | 3.51 | 73.88 | 3.38 | 178.31 | 75.22 | 1.00 | 2.99 |
| 2015 | 2 | B | Bulindi | 6.01 | 68.22 | 0.69 | . | . | 1.00 | 3.09 |
| 2015 | 2 | B | Ikulwe | 5.16 | 63.13 | 0.50 | 246.82 | 125.30 | 0.96 | 3.11 |
| 2015 | 2 | B | Masaka | 7.26 | 70.09 | 1.69 | . | . | 0.95 | 2.23 |
| 2015 | 2 | B | Namulonge | 5.76 | 65.30 | -0.66 | 247.66 | 118.73 | 1.02 | 2.91 |
| 2015 | 2 | B | Ngetta | 1.26 | 64.28 | 4.42 | . | . | . | 2.70 |
| 2015 | 2 | B | Serere | 3.39 | 64.11 | 0.91 | 189.04 | 91.11 | 0.89 | 3.57 |
| 2015 | 3 | B | Bulegeni | 2.87 | 73.67 | 3.04 | 202.91 | 91.12 | 1.00 | 3.71 |
| 2015 | 3 | B | Bulindi | 5.17 | 69.20 | 0.89 | 204.70 | 112.51 | . | 2.85 |
| 2015 | 3 | B | Ikulwe | 4.88 | 63.78 | 0.51 | 254.53 | 134.88 | 1.03 | 2.63 |
| 2015 | 3 | B | Masaka | 4.68 | 71.40 | 1.88 | . | 30.12 | . | 2.10 |
| 2015 | 3 | B | Namulonge | 5.05 | 65.54 | -0.24 | 244.37 | 117.62 | 1.05 | 3.20 |
| 2015 | 3 | B | Ngetta | 1.37 | 62.96 | 2.97 | 185.49 | 109.16 | . | 2.42 |
| 2015 | 3 | B | Serere | 3.68 | 64.03 | 1.05 | . | 93.38 | . | 3.20 |
| 2016 | 1 | B | Bulindi | 6.75 | 66.19 | 1.75 | 200.24 | 88.39 | 1.10 | 2.61 |
| 2016 | 1 | B | Masaka | 3.29 | 76.74 | 1.91 | . | . | 1.03 | 2.48 |
| 2016 | 1 | B | Namulonge | 1.66 | 66.72 | 0.76 | . | . | 1.01 | 2.86 |
| 2016 | 1 | B | Serere | 1.49 | 61.32 | 1.24 | 166.43 | 79.48 | 1.06 | 2.25 |
| 2016 | 2 | B | Bulindi | 5.17 | 66.81 | 1.94 | 189.48 | 79.42 | 1.06 | 2.84 |
| 2016 | 2 | B | Masaka | 3.91 | 75.50 | 4.13 | . | . | 1.05 | 3.24 |
| 2016 | 3 | B | Abii | 2.76 | 70.63 | -1.06 | . | . | 0.88 | 2.82 |
| 2016 | 3 | B | Bulindi | 6.62 | 65.67 | 2.13 | 202.68 | 90.77 | 1.00 | 2.61 |
| 2016 | 3 | B | Masaka | 3.64 | 76.35 | 2.18 | . | . | 0.98 | 2.58 |
| 2017 | 1 | A | Abii | 3.22 | 68.62 | 4.09 | 149.93 | 58.81 | 0.94 | 3.55 |
| 2017 | 1 | A | Bulindi | 7.04 | 61.82 | 0.97 | 185.80 | 86.08 | 0.97 | 3.47 |
| 2017 | 1 | A | Ngetta | 5.96 | 58.34 | 1.65 | 236.94 | 126.64 | 1.02 | 2.94 |
| 2017 | 1 | A | Serere | 3.57 | 61.96 | 1.39 | 200.87 | 101.84 | . | 2.75 |
| 2017 | 2 | A | Abii | 3.00 | 68.14 | 4.21 |  |  | . | 3.14 |
| 2017 | 2 | A | Bulegeni | 3.04 | 69.39 | 0.30 | . | . | 0.60 | 2.80 |
| 2017 | 2 | A | Bulindi | 5.87 | 61.09 | 0.69 | 180.41 | 85.70 | 0.99 | 2.41 |
| 2017 | 2 | A | Ngetta | 6.57 | 59.30 | 1.86 | 226.95 | 119.31 | 0.87 | 2.57 |
| 2017 | 2 | A | Serere | 3.07 | 62.16 | 1.42 | 198.69 | 102.50 | . | 2.52 |
| 2017 | 3 | A | Abii | 2.31 | 70.36 | 4.24 | . | . | 0.84 | 3.00 |
| 2017 | 3 | A | Bulegeni | 4.47 | 70.13 | 0.04 | . | . | 0.67 | 2.11 |
| 2017 | 3 | A | Bulindi | 6.89 | 63.28 | 1.93 | 184.93 | 88.09 | 1.00 | 3.05 |
| 2017 | 3 | A | Ngetta | 5.82 | 61.09 | 2.05 | 209.58 | 111.22 | 0.82 | 2.58 |
| 2017 | 3 | A | Serere | 3.83 | 62.88 | 1.41 | 206.93 | 110.09 | . | 2.60 |
| 2018 | 1 | A | Bulegeni | 3.55 | 69.40 | 1.83 | . | . | 1.10 | 2.60 |
| 2018 | 1 | A | Bulindi | 3.03 | 60.73 | 3.40 | 189.87 | 83.33 | 1.05 | 2.97 |
| 2018 | 1 | A | Namulonge | 1.98 | 70.58 | 2.56 | 134.69 | 47.96 | 1.11 | 1.54 |
| 2018 | 1 | A | Ngetta | 3.01 | 65.07 | 2.40 | 167.00 | 66.47 | 0.97 | 2.59 |
| 2018 | 1 | A | Serere | 2.60 | 66.63 | 1.14 | 165.53 | 64.07 | 1.02 | . |
| 2018 | 3 | A | Bulegeni | 6.14 | 66.27 | 1.63 | . | . | 1.09 | 2.38 |
| 2018 | 3 | A | Bulindi | 6.34 | 60.98 | 1.60 | 203.39 | 96.50 | 1.01 | 2.68 |
| 2018 | 3 | A | Ikulwe | 2.80 | 64.18 | 1.24 |  | . | . | 2.78 |
| 2018 | 3 | A | Ngetta | 7.70 | 72.71 | 3.54 | 207.07 | 99.60 | 1.07 | 3.12 |
| 2018 | 3 | A | Serere | 3.33 | 69.46 | 1.36 | 163.30 | 60.55 | 0.98 | 2.48 |
| 2019 | 1 | B | Bulegeni | 3.64 | . | . | . | . | . | 1.34 |
| 2019 | 1 | B | Bulindi | 3.74 | . | . | . | . | 1.06 | 1.93 |
| 2019 | 1 | B | Ikulwe | 3.97 | . | . | . | . | 0.96 | 1.75 |
| 2019 | 1 | B | Namulonge | 1.57 | . | . | . | . | 0.63 | 1.38 |
| 2019 | 1 | B | Ngetta | 2.92 | . | . | . | . | . | 1.38 |
| 2019 | 1 | B | Serere | 3.97 | . | . | . | . | 0.96 | 1.75 |
| 2020 | 3 | A | Bulegeni | 6.31 | 72.14 | 1.92 | 216.89 | 103.71 | 1.10 | 3.44 |
| 2020 | 3 | A | Bulindi | 5.36 | 62.75 | 1.52 | 195.84 | 99.41 | 1.04 | 2.61 |
| 2020 | 3 | A | Ikulwe | 3.76 | 61.76 | 1.94 | 188.97 | 80.46 | 1.08 | 2.31 |
| 2020 | 3 | A | Namulonge | 3.41 | 68.46 | 3.14 | 133.44 | 61.70 | 1.03 | 2.35 |
| 2020 | 3 | A | Serere | 7.40 | 59.60 | 0.76 | 249.16 | 126.60 | 1.05 | 2.68 |

Supplemental Table B. Name source and testing history of the 419 maize lines in the 2008-2020 National Performance Trial conducted by the National Agriculture Research Organization of Uganda

| **#** | **Entry** | **Source** | **First Year Tested** | **Last Year Tested** |
| --- | --- | --- | --- | --- |
| 1 | 12 | Check | 2015 | 2020 |
| 2 | 5 | Check | 2019 | 2019 |
| 3 | 5 | Check | 2009 | 2020 |
| 4 | 6 | Check | 2009 | 2011 |
| 5 | 4 | Check | 2019 | 2019 |
| 6 | 8 | Check | 2008 | 2017 |
| 7 | 15 | Check | 2011 | 2014 |
| 8 | 18 | Check | 2012 | 2017 |
| 9 | 4 | Check | 2015 | 2015 |
| 10 | 7 | Check | 2010 | 2018 |
| 11 | 14 | Check | 2019 | 2019 |
| 12 | 21 | Check | 2015 | 2015 |
| 13 | 4 | CIMMYT | 2019 | 2019 |
| 14 | 4 | CIMMYT | 2018 | 2020 |
| 15 | 9 | CIMMYT | 2012 | 2013 |
| 16 | 4 | CIMMYT | 2012 | 2013 |
| 17 | 10 | CIMMYT | 2012 | 2013 |
| 18 | 15 | CIMMYT | 2012 | 2013 |
| 19 | 5 | CIMMYT | 2012 | 2013 |
| 20 | 15 | CIMMYT | 2012 | 2013 |
| 21 | 6 | CIMMYT | 2018 | 2020 |
| 22 | 15 | CIMMYT | 2015 | 2018 |
| 23 | 6 | CIMMYT | 2015 | 2020 |
| 24 | 12 | CIMMYT | 2015 | 2020 |
| 25 | 5 | CIMMYT | 2015 | 2015 |
| 26 | 5 | CIMMYT | 2015 | 2020 |
| 27 | 6 | CIMMYT | 2015 | 2020 |
| 28 | 4 | CIMMYT | 2016 | 2016 |
| 29 | 8 | CIMMYT | 2016 | 2016 |
| 30 | 15 | CIMMYT | 2016 | 2016 |
| 31 | 18 | CIMMYT | 2016 | 2016 |
| 32 | 4 | CIMMYT | 2016 | 2016 |
| 33 | 7 | CIMMYT | 2016 | 2020 |
| 34 | 14 | CIMMYT | 2016 | 2016 |
| 35 | 21 | CIMMYT | 2016 | 2016 |
| 36 | 4 | CIMMYT | 2016 | 2020 |
| 37 | 4 | CIMMYT | 2016 | 2020 |
| 38 | 9 | CIMMYT | 2018 | 2020 |
| 39 | 4 | CIMMYT | 2016 | 2018 |
| 40 | 10 | CIMMYT | 2016 | 2018 |
| 41 | 15 | CIMMYT | 2016 | 2018 |
| 42 | 5 | CIMMYT | 2016 | 2017 |
| 43 | 15 | CIMMYT | 2016 | 2018 |
| 44 | 6 | CIMMYT | 2016 | 2018 |
| 45 | 15 | CIMMYT | 2017 | 2018 |
| 46 | 6 | CIMMYT | 2008 | 2008 |
| 47 | 12 | CIMMYT | 2008 | 2008 |
| 48 | 5 | CIMMYT | 2008 | 2008 |
| 49 | 5 | CIMMYT | 2008 | 2008 |
| 50 | 6 | CIMMYT | 2008 | 2008 |
| 51 | 4 | CIMMYT | 2008 | 2008 |
| 52 | 8 | CIMMYT | 2011 | 2012 |
| 53 | 15 | CIMMYT | 2012 | 2012 |
| 54 | 18 | CIMMYT | 2019 | 2019 |
| 55 | 4 | CIMMYT | 2019 | 2019 |
| 56 | 7 | CIMMYT | 2019 | 2019 |
| 57 | 14 | CIMMYT | 2019 | 2019 |
| 58 | 21 | CIMMYT | 2019 | 2019 |
| 59 | 4 | CIMMYT | 2019 | 2019 |
| 60 | 4 | CIMMYT | 2015 | 2015 |
| 61 | 9 | CIMMYT | 2015 | 2015 |
| 62 | 4 | CIMMYT | 2015 | 2015 |
| 63 | 10 | CIMMYT | 2015 | 2015 |
| 64 | 15 | CIMMYT | 2015 | 2015 |
| 65 | 5 | CIMMYT | 2015 | 2015 |
| 66 | 15 | CIMMYT | 2015 | 2015 |
| 67 | 6 | CIMMYT | 2008 | 2008 |
| 68 | 15 | CIMMYT | 2019 | 2019 |
| 69 | 6 | CIMMYT | 2019 | 2019 |
| 70 | 12 | CIMMYT | 2019 | 2019 |
| 71 | 5 | CIMMYT | 2008 | 2008 |
| 72 | 5 | CIMMYT | 2008 | 2008 |
| 73 | 6 | CIMMYT | 2019 | 2019 |
| 74 | 4 | CIMMYT | 2019 | 2019 |
| 75 | 8 | CIMMYT | 2019 | 2019 |
| 76 | 15 | CIMMYT | 2019 | 2019 |
| 77 | 18 | CIMMYT | 2019 | 2019 |
| 78 | 4 | CIMMYT | 2019 | 2019 |
| 79 | 7 | CIMMYT | 2019 | 2019 |
| 80 | 14 | CIMMYT | 2019 | 2019 |
| 81 | 21 | CIMMYT | 2019 | 2019 |
| 82 | 4 | CIMMYT | 2019 | 2019 |
| 83 | 4 | CIMMYT | 2019 | 2019 |
| 84 | 9 | CIMMYT | 2019 | 2019 |
| 85 | 4 | CIMMYT | 2019 | 2019 |
| 86 | 10 | CIMMYT | 2019 | 2019 |
| 87 | 15 | CIMMYT | 2019 | 2019 |
| 88 | 5 | CIMMYT | 2017 | 2018 |
| 89 | 15 | CIMMYT | 2017 | 2017 |
| 90 | 6 | CIMMYT | 2017 | 2018 |
| 91 | 15 | CIMMYT | 2017 | 2017 |
| 92 | 6 | CIMMYT | 2012 | 2013 |
| 93 | 12 | CIMMYT | 2012 | 2013 |
| 94 | 5 | CIMMYT | 2012 | 2013 |
| 95 | 5 | CIMMYT | 2012 | 2013 |
| 96 | 6 | CIMMYT | 2012 | 2013 |
| 97 | 4 | CIMMYT | 2012 | 2013 |
| 98 | 8 | CIMMYT | 2012 | 2013 |
| 99 | 15 | CIMMYT | 2012 | 2013 |
| 100 | 18 | CIMMYT | 2015 | 2016 |
| 101 | 4 | CIMMYT | 2015 | 2018 |
| 102 | 7 | CIMMYT | 2015 | 2016 |
| 103 | 14 | CIMMYT | 2015 | 2016 |
| 104 | 21 | CIMMYT | 2015 | 2016 |
| 105 | 4 | CIMMYT | 2015 | 2016 |
| 106 | 4 | CIMMYT | 2015 | 2015 |
| 107 | 9 | CIMMYT | 2015 | 2016 |
| 108 | 4 | CIMMYT | 2015 | 2015 |
| 109 | 10 | CIMMYT | 2015 | 2015 |
| 110 | 15 | CIMMYT | 2015 | 2016 |
| 111 | 5 | CIMMYT | 2015 | 2015 |
| 112 | 15 | CIMMYT | 2015 | 2015 |
| 113 | 6 | CIMMYT | 2015 | 2015 |
| 114 | 15 | CIMMYT | 2015 | 2015 |
| 115 | 6 | CIMMYT | 2015 | 2015 |
| 116 | 12 | CIMMYT | 2015 | 2015 |
| 117 | 5 | CIMMYT | 2015 | 2015 |
| 118 | 5 | CIMMYT | 2015 | 2015 |
| 119 | 6 | CIMMYT | 2015 | 2015 |
| 120 | 4 | CIMMYT | 2015 | 2015 |
| 121 | 8 | CIMMYT | 2015 | 2015 |
| 122 | 15 | CIMMYT | 2015 | 2015 |
| 123 | 18 | CIMMYT | 2015 | 2016 |
| 124 | 4 | CIMMYT | 2015 | 2016 |
| 125 | 7 | CIMMYT | 2015 | 2017 |
| 126 | 14 | CIMMYT | 2015 | 2015 |
| 127 | 21 | CIMMYT | 2015 | 2015 |
| 128 | 4 | CIMMYT | 2015 | 2015 |
| 129 | 4 | CIMMYT | 2015 | 2017 |
| 130 | 9 | CIMMYT | 2016 | 2017 |
| 131 | 4 | CIMMYT | 2015 | 2017 |
| 132 | 10 | CIMMYT | 2016 | 2017 |
| 133 | 15 | CIMMYT | 2015 | 2017 |
| 134 | 5 | CIMMYT | 2015 | 2015 |
| 135 | 15 | CIMMYT | 2015 | 2015 |
| 136 | 6 | CIMMYT | 2015 | 2015 |
| 137 | 15 | CIMMYT | 2015 | 2015 |
| 138 | 6 | CIMMYT | 2015 | 2017 |
| 139 | 12 | CIMMYT | 2015 | 2015 |
| 140 | 5 | CIMMYT | 2015 | 2017 |
| 141 | 5 | CIMMYT | 2015 | 2016 |
| 142 | 6 | CIMMYT | 2015 | 2016 |
| 143 | 4 | CIMMYT | 2017 | 2018 |
| 144 | 8 | CIMMYT | 2017 | 2018 |
| 145 | 15 | CIMMYT | 2017 | 2018 |
| 146 | 18 | CIMMYT | 2017 | 2018 |
| 147 | 4 | CIMMYT | 2015 | 2015 |
| 148 | 7 | CIMMYT | 2015 | 2015 |
| 149 | 14 | CIMMYT | 2015 | 2015 |
| 150 | 21 | CIMMYT | 2015 | 2015 |
| 151 | 4 | CIMMYT | 2015 | 2015 |
| 152 | 4 | CIMMYT | 2015 | 2015 |
| 153 | 9 | CIMMYT | 2015 | 2015 |
| 154 | 4 | CIMMYT | 2015 | 2015 |
| 155 | 10 | CIMMYT | 2015 | 2015 |
| 156 | 15 | CIMMYT | 2015 | 2015 |
| 157 | 5 | CIMMYT | 2015 | 2015 |
| 158 | 15 | CIMMYT | 2015 | 2015 |
| 159 | 6 | CIMMYT | 2015 | 2015 |
| 160 | 15 | CIMMYT | 2015 | 2015 |
| 161 | 6 | CIMMYT | 2015 | 2015 |
| 162 | 12 | CIMMYT | 2015 | 2015 |
| 163 | 5 | CIMMYT | 2015 | 2015 |
| 164 | 5 | CIMMYT | 2015 | 2015 |
| 165 | 6 | CIMMYT | 2017 | 2017 |
| 166 | 4 | CIMMYT | 2017 | 2018 |
| 167 | 8 | CIMMYT | 2017 | 2018 |
| 168 | 15 | CIMMYT | 2017 | 2018 |
| 169 | 18 | CIMMYT | 2017 | 2018 |
| 170 | 4 | CIMMYT | 2017 | 2018 |
| 171 | 7 | CIMMYT | 2017 | 2018 |
| 172 | 14 | CIMMYT | 2017 | 2017 |
| 173 | 21 | CIMMYT | 2016 | 2017 |
| 174 | 4 | CIMMYT | 2016 | 2017 |
| 175 | 4 | CIMMYT | 2016 | 2017 |
| 176 | 9 | CIMMYT | 2017 | 2018 |
| 177 | 4 | CIMMYT | 2017 | 2017 |
| 178 | 10 | CIMMYT | 2017 | 2018 |
| 179 | 15 | CIMMYT | 2017 | 2018 |
| 180 | 5 | CIMMYT | 2017 | 2018 |
| 181 | 15 | CIMMYT | 2017 | 2018 |
| 182 | 6 | CIMMYT | 2017 | 2018 |
| 183 | 15 | CIMMYT | 2017 | 2018 |
| 184 | 6 | CIMMYT | 2017 | 2018 |
| 185 | 12 | CIMMYT | 2017 | 2018 |
| 186 | 5 | CIMMYT | 2017 | 2017 |
| 187 | 5 | CIMMYT | 2017 | 2017 |
| 188 | 6 | CIMMYT | 2017 | 2017 |
| 189 | 4 | CIMMYT | 2017 | 2018 |
| 190 | 8 | CIMMYT | 2017 | 2018 |
| 191 | 15 | CIMMYT | 2017 | 2017 |
| 192 | 18 | CIMMYT | 2017 | 2017 |
| 193 | 4 | CIMMYT | 2017 | 2017 |
| 194 | 7 | CIMMYT | 2017 | 2017 |
| 195 | 14 | CIMMYT | 2017 | 2017 |
| 196 | 21 | CIMMYT | 2012 | 2012 |
| 197 | 4 | CIMMYT | 2012 | 2012 |
| 198 | 4 | CIMMYT | 2012 | 2012 |
| 199 | 9 | CIMMYT | 2012 | 2012 |
| 200 | 4 | CIMMYT | 2012 | 2012 |
| 201 | 10 | CIMMYT | 2012 | 2012 |
| 202 | 15 | CIMMYT | 2012 | 2012 |
| 203 | 5 | CIMMYT | 2012 | 2012 |
| 204 | 15 | CIMMYT | 2012 | 2012 |
| 205 | 6 | CIMMYT | 2012 | 2012 |
| 206 | 15 | CIMMYT | 2012 | 2012 |
| 207 | 6 | CIMMYT | 2012 | 2012 |
| 208 | 12 | CIMMYT | 2012 | 2012 |
| 209 | 5 | CIMMYT | 2012 | 2012 |
| 210 | 5 | CIMMYT | 2012 | 2012 |
| 211 | 6 | CIMMYT | 2012 | 2012 |
| 212 | 4 | CIMMYT | 2017 | 2017 |
| 213 | 8 | CIMMYT | 2017 | 2017 |
| 214 | 15 | CIMMYT | 2017 | 2017 |
| 215 | 18 | CIMMYT | 2017 | 2017 |
| 216 | 4 | CIMMYT | 2017 | 2017 |
| 217 | 7 | CIMMYT | 2017 | 2017 |
| 218 | 14 | CIMMYT | 2017 | 2017 |
| 219 | 21 | CIMMYT | 2017 | 2017 |
| 220 | 4 | CIMMYT | 2017 | 2017 |
| 221 | 4 | CIMMYT | 2017 | 2017 |
| 222 | 9 | CIMMYT | 2017 | 2017 |
| 223 | 4 | CIMMYT | 2017 | 2017 |
| 224 | 10 | NARO | 2010 | 2010 |
| 225 | 15 | NARO | 2012 | 2013 |
| 226 | 5 | NARO | 2012 | 2013 |
| 227 | 15 | NARO | 2019 | 2019 |
| 228 | 6 | NARO | 2019 | 2019 |
| 229 | 15 | NARO | 2019 | 2019 |
| 230 | 6 | NARO | 2019 | 2019 |
| 231 | 12 | NARO | 2019 | 2019 |
| 232 | 5 | NARO | 2014 | 2015 |
| 233 | 5 | NARO | 2014 | 2014 |
| 234 | 6 | NARO | 2014 | 2015 |
| 235 | 4 | NARO | 2014 | 2014 |
| 236 | 8 | NARO | 2014 | 2014 |
| 237 | 15 | NARO | 2014 | 2014 |
| 238 | 18 | NARO | 2014 | 2015 |
| 239 | 4 | NARO | 2014 | 2015 |
| 240 | 7 | NARO | 2014 | 2014 |
| 241 | 14 | NARO | 2014 | 2015 |
| 242 | 21 | NARO | 2014 | 2014 |
| 243 | 4 | NARO | 2014 | 2014 |
| 244 | 4 | NARO | 2014 | 2014 |
| 245 | 9 | NARO | 2014 | 2014 |
| 246 | 4 | NARO | 2012 | 2013 |
| 247 | 10 | NARO | 2012 | 2013 |
| 248 | 15 | NARO | 2009 | 2009 |
| 249 | 5 | NARO | 2009 | 2010 |
| 250 | 15 | NARO | 2016 | 2016 |
| 251 | 6 | NARO | 2016 | 2016 |
| 252 | 15 | NARO | 2008 | 2008 |
| 253 | 6 | NARO | 2008 | 2008 |
| 254 | 12 | NARO | 2008 | 2008 |
| 255 | 5 | NARO | 2008 | 2008 |
| 256 | 5 | NARO | 2008 | 2008 |
| 257 | 6 | NARO | 2008 | 2008 |
| 258 | 4 | NARO | 2009 | 2009 |
| 259 | 8 | NARO | 2011 | 2013 |
| 260 | 15 | NARO | 2011 | 2013 |
| 261 | 18 | NARO | 2011 | 2013 |
| 262 | 4 | NARO | 2009 | 2009 |
| 263 | 7 | NARO | 2011 | 2013 |
| 264 | 14 | NARO | 2011 | 2013 |
| 265 | 21 | NARO | 2011 | 2013 |
| 266 | 4 | NARO | 2008 | 2008 |
| 267 | 4 | NARO | 2008 | 2008 |
| 268 | 9 | NARO | 2008 | 2008 |
| 269 | 4 | NARO | 2008 | 2008 |
| 270 | 10 | NARO | 2008 | 2008 |
| 271 | 15 | NARO | 2008 | 2008 |
| 272 | 5 | NARO | 2008 | 2008 |
| 273 | 15 | NARO | 2008 | 2008 |
| 274 | 6 | NARO | 2008 | 2008 |
| 275 | 15 | NARO | 2008 | 2008 |
| 276 | 6 | NARO | 2008 | 2008 |
| 277 | 12 | NARO | 2008 | 2008 |
| 278 | 5 | NARO | 2008 | 2008 |
| 279 | 5 | NARO | 2009 | 2009 |
| 280 | 6 | NARO | 2011 | 2013 |
| 281 | 4 | NARO | 2011 | 2013 |
| 282 | 8 | NARO | 2011 | 2013 |
| 283 | 15 | NARO | 2011 | 2013 |
| 284 | 18 | NARO | 2009 | 2009 |
| 285 | 4 | NARO | 2010 | 2010 |
| 286 | 7 | NARO | 2010 | 2010 |
| 287 | 14 | NARO | 2017 | 2017 |
| 288 | 21 | NARO | 2017 | 2017 |
| 289 | 4 | NARO | 2017 | 2017 |
| 290 | 4 | NARO | 2017 | 2017 |
| 291 | 9 | NARO | 2017 | 2017 |
| 292 | 4 | NARO | 2017 | 2017 |
| 293 | 10 | NARO | 2017 | 2017 |
| 294 | 15 | NARO | 2017 | 2017 |
| 295 | 5 | NARO | 2017 | 2017 |
| 296 | 15 | NARO | 2017 | 2017 |
| 297 | 6 | NARO | 2017 | 2017 |
| 298 | 15 | Private | 2015 | 2015 |
| 299 | 6 | Private | 2015 | 2016 |
| 300 | 12 | Private | 2015 | 2016 |
| 301 | 5 | Private | 2015 | 2016 |
| 302 | 5 | Private | 2015 | 2016 |
| 303 | 6 | Private | 2010 | 2010 |
| 304 | 4 | Private | 2010 | 2010 |
| 305 | 8 | Private | 2017 | 2018 |
| 306 | 15 | Private | 2017 | 2018 |
| 307 | 18 | Private | 2017 | 2017 |
| 308 | 4 | Private | 2017 | 2017 |
| 309 | 7 | Private | 2017 | 2017 |
| 310 | 14 | Private | 2018 | 2020 |
| 311 | 21 | Private | 2020 | 2020 |
| 312 | 4 | Private | 2016 | 2017 |
| 313 | 4 | Private | 2017 | 2017 |
| 314 | 9 | Private | 2017 | 2017 |
| 315 | 4 | Private | 2016 | 2017 |
| 316 | 10 | Private | 2018 | 2018 |
| 317 | 15 | Private | 2018 | 2018 |
| 318 | 5 | Private | 2014 | 2015 |
| 319 | 15 | Private | 2014 | 2015 |
| 320 | 6 | Private | 2015 | 2015 |
| 321 | 15 | Private | 2012 | 2013 |
| 322 | 6 | Private | 2010 | 2018 |
| 323 | 12 | Private | 2011 | 2012 |
| 324 | 5 | Private | 2009 | 2009 |
| 325 | 5 | Private | 2015 | 2015 |
| 326 | 6 | Private | 2015 | 2015 |
| 327 | 4 | Private | 2008 | 2008 |
| 328 | 8 | Private | 2011 | 2011 |
| 329 | 15 | Private | 2017 | 2018 |
| 330 | 18 | Private | 2014 | 2015 |
| 331 | 4 | Private | 2014 | 2015 |
| 332 | 7 | Private | 2014 | 2015 |
| 333 | 14 | Private | 2014 | 2015 |
| 334 | 21 | Private | 2014 | 2015 |
| 335 | 4 | Private | 2015 | 2017 |
| 336 | 4 | Private | 2015 | 2015 |
| 337 | 9 | Private | 2015 | 2015 |
| 338 | 4 | Private | 2015 | 2015 |
| 339 | 10 | Private | 2017 | 2017 |
| 340 | 15 | Private | 2015 | 2017 |
| 341 | 5 | Private | 2015 | 2016 |
| 342 | 15 | Private | 2018 | 2018 |
| 343 | 6 | Private | 2018 | 2018 |
| 344 | 15 | Private | 2018 | 2018 |
| 345 | 6 | Private | 2014 | 2017 |
| 346 | 12 | Private | 2014 | 2014 |
| 347 | 5 | Private | 2009 | 2010 |
| 348 | 5 | Private | 2009 | 2010 |
| 349 | 6 | Private | 2009 | 2010 |
| 350 | 4 | Private | 2009 | 2010 |
| 351 | 8 | Private | 2017 | 2017 |
| 352 | 15 | Private | 2015 | 2015 |
| 353 | 18 | Private | 2016 | 2018 |
| 354 | 4 | Private | 2017 | 2017 |
| 355 | 7 | Private | 2015 | 2016 |
| 356 | 14 | Private | 2015 | 2015 |
| 357 | 21 | Private | 2009 | 2010 |
| 358 | 4 | Private | 2015 | 2015 |
| 359 | 4 | Private | 2009 | 2010 |
| 360 | 9 | Private | 2017 | 2017 |
| 361 | 4 | Private | 2017 | 2017 |
| 362 | 10 | Private | 2012 | 2017 |
| 363 | 15 | Private | 2011 | 2012 |
| 364 | 5 | Private | 2017 | 2018 |
| 365 | 15 | Private | 2017 | 2017 |
| 366 | 6 | Private | 2017 | 2017 |
| 367 | 15 | Private | 2018 | 2018 |
| 368 | 6 | Private | 2008 | 2012 |
| 369 | 12 | Private | 2015 | 2018 |
| 370 | 5 | Private | 2015 | 2015 |
| 371 | 5 | Private | 2015 | 2018 |
| 372 | 6 | Private | 2017 | 2020 |
| 373 | 4 | Private | 2017 | 2017 |
| 374 | 8 | Private | 2015 | 2015 |
| 375 | 15 | Private | 2015 | 2020 |
| 376 | 18 | Private | 2015 | 2016 |
| 377 | 4 | Private | 2015 | 2016 |
| 378 | 7 | Private | 2015 | 2016 |
| 379 | 14 | Private | 2015 | 2015 |
| 380 | 21 | Private | 2017 | 2017 |
| 381 | 4 | Private | 2015 | 2015 |
| 382 | 4 | Private | 2017 | 2017 |
| 383 | 9 | Private | 2015 | 2016 |
| 384 | 4 | Private | 2015 | 2016 |
| 385 | 10 | Private | 2015 | 2018 |
| 386 | 15 | Private | 2020 | 2020 |
| 387 | 5 | Private | 2018 | 2018 |
| 388 | 15 | Private | 2017 | 2018 |
| 389 | 6 | Private | 2017 | 2018 |
| 390 | 15 | Private | 2010 | 2010 |
| 391 | 6 | Private | 2008 | 2013 |
| 392 | 12 | Private | 2008 | 2013 |
| 393 | 5 | Private | 2011 | 2015 |
| 394 | 5 | Private | 2015 | 2015 |
| 395 | 6 | Private | 2017 | 2018 |
| 396 | 4 | Private | 2011 | 2012 |
| 397 | 8 | Private | 2011 | 2011 |
| 398 | 15 | Private | 2018 | 2018 |
| 399 | 18 | Private | 2008 | 2008 |
| 400 | 4 | Private | 2011 | 2012 |
| 401 | 7 | Private | 2012 | 2018 |
| 402 | 14 | Private | 2011 | 2018 |
| 403 | 21 | Private | 2017 | 2018 |
| 404 | 4 | Private | 2014 | 2015 |
| 405 | 4 | Private | 2015 | 2015 |
| 406 | 9 | Private | 2017 | 2018 |
| 407 | 4 | Private | 2017 | 2018 |
| 408 | 10 | Private | 2017 | 2018 |
| 409 | 15 | Private | 2017 | 2018 |
| 410 | 5 | Private | 2017 | 2017 |
| 411 | 15 | Private | 2011 | 2012 |
| 412 | 6 | Private | 2020 | 2020 |
| 413 | 15 | Private | 2020 | 2020 |
| 414 | 6 | Private | 2020 | 2020 |
| 415 | 12 | Private | 2020 | 2020 |
| 416 | 5 | Private | 2020 | 2020 |
| 417 | 5 | Private | 2020 | 2020 |
| 418 | 6 | Private | 2020 | 2020 |
| 419 | 4 | Private | 2020 | 2020 |
